# Mesenchymal Transformation: The Rosetta Stone of Glioblastoma Pathogenesis and Therapy Resistance

**DOI:** 10.1002/advs.202002015

**Published:** 2020-09-28

**Authors:** Zulfikar Azam, Shing‐Shun Tony TO, Bakhos A. Tannous

**Affiliations:** ^1^ Experimental Therapeutics and Molecular Imaging Unit Department of Neurology Neuro‐Oncology Division Massachusetts General Hospital and Harvard Medical School Boston MA 02129 USA; ^2^ Department of Health Technology and Informatics The Hong Kong Polytechnic University Hong Kong 999077 China

**Keywords:** clinical outcome, glioblastoma (GBM), mesenchymal transition, molecular subtypes, therapy responses, tumor microenvironment

## Abstract

Despite decades of research, glioblastoma (GBM) remains invariably fatal among all forms of cancers. The high level of inter‐ and intratumoral heterogeneity along with its biological location, the brain, are major barriers against effective treatment. Molecular and single cell analysis identifies different molecular subtypes with varying prognosis, while multiple subtypes can reside in the same tumor. Cellular plasticity among different subtypes in response to therapies or during recurrence adds another hurdle in the treatment of GBM. This phenotypic shift is induced and sustained by activation of several pathways within the tumor itself, or microenvironmental factors. In this review, the dynamic nature of cellular shifts in GBM and how the tumor (immune) microenvironment shapes this process leading to therapeutic resistance, while highlighting emerging tools and approaches to study this dynamic double‐edged sword are discussed.

## Graphical abstract

This review summarizes the dynamic nature of cellular shift in glioblastoma and how the tumor (immune) microenvironment shapes this process leading to therapeutic resistance, while highlighting emerging tools and approaches to study this dynamic double‐edged sword.

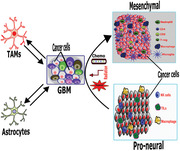

## 1 Introduction

Gliomas account for over 80% of all fatal central nervous system (CNS) malignancy^[^ ^1^ ^]^ and are the leading cause of cancer‐related mortality among children^[^ ^2^ ^]^ and adults aged below 34 years.^[^ ^3^ ^]^ Based on histological properties, gliomas are classified into four classes (grades I–IV), in which grade IV, commonly referred to as glioblastoma (GBM), is the most malignant form with an overall mean survival rate of 12–17 months from diagnosis.^[^ ^4^, ^5^, ^6^, ^7^, ^8^, ^9^ ^]^ GBMs are highly heterogeneous in nature and tend to infiltrate surrounding areas making complete surgical resection (first line therapy) practically impossible.^[^ ^10^ ^]^ The second line treatment for GBM, consisting of radiotherapy with concomitant temozolomide (TMZ) chemotherapy, also fails partly due to intrinsic and/or acquired radio and chemoresistance.^[10]^ The histological features of glioma (necrosis, cellularity, vascular proliferation, and mitotic figures) do not reflect the treatment failure and poor clinical outcome.^[^ ^11^ ^]^ Thus, researchers now are focusing on the molecular features of GBM rather than its histological properties. Large‐scale genomic analysis has subgrouped GBM into four main classes, classical, pro‐neural (PN), mesenchymal (MES), and neural, although later studies showed that the neural subtype is simply a contamination with nontumor cells.^[^ ^12^, ^13^ ^]^ Each of these subtypes showed a different degree of sensitivity to therapy, while prognosis corresponds to their molecular and genetic signature.^[^ ^14^ ^]^ For example, tumors with MES feature tend to have worst prognosis compared to other subtypes (PN and classical).^[^ ^14^ ^]^ In addition, over the course of the disease progression, GBMs can shift their subtypes from one to another^[^ ^14^ ^]^ and it is believed that the pro‐neural‐to‐mesenchymal transition (PMT) is most prominent.^[^ ^15^ ^]^ Segerman et al. found that, upon radiation and chemotherapy, PN tumors shift to MES subtype ultimately leading to chemo and radioresistance.^[^ ^16^ ^]^ This PMT is synonymous to epithelial‐to‐mesenchymal transition (EMT) in solid tumors of epithelial origin.^[^ ^17^ ^]^ Because of the believed glial origin of GBM, this process is typically referred to as glial‐mesenchymal transition, or simply mesenchymal transformation/transition/transdifferentiation, and more commonly PMT, since this transition is typically observed in the pro‐neural subtype.^[^ ^11^, ^18^ ^]^ EMT and PMT share similar patterns of cellular properties, pathological features, and molecular marker changes. EMT occurs when tumor cells lose their epithelial characteristics (cell–cell adhesion and polarity) and possess a more aggressive mesenchymal characteristic.^[^ ^19^ ^]^ Although EMT plays a crucial role in embryonic development and wound healing processes,^[^ ^19^ ^]^ it was proved to be of significance to tumor cell migration, invasion, metastasis, and resistance to conventional radio and chemotherapy.^[^ ^20^ ^]^ EMT is also crucial for the development and maintenance of cancer stem cell (CSC), a small niche of cells believed to be responsible for tumor initiation, recurrence, and resistance to conventional treatment.^[^ ^20^ ^]^ In GBM, this cell subset is referred to as glioma stem cells (GSCs) with neural stem‐like properties. External stimuli such as growth factors, cytokines, and hypoxic environment induce a vast array of signaling pathways and associated transcription factors (SNAI1, SLUG, ZEB, and TWIST) leading to EMT transition.^[^ ^21^ ^]^ The same molecular events are also involved in mesenchymal transition in GBM leading to increased tumor aggressiveness and acquired drug resistance. In this context, understanding key players that drive mesenchymal transformation and their relationship with drug resistance and poor prognosis is critical to develop effective therapies against this aggressive disease. In this review, we will discuss recent advances and challenges in studying GBM mesenchymal transformation, while highlighting state‐of‐the‐art tools to study these processes.

## 2 Molecular Characteristics of Glioblastoma Subtypes

One of the main and probably the most important driving factors for treatment failure in GBM is the inter‐ and intratumoral heterogeneity. Molecular subtypes of GBM first reported by Phillips et al., classified gliomas into three subclasses, namely, pro‐neural, proliferative, and mesenchymal based on 35 genes.^[^ ^22^ ^]^ In this study, they analyzed genetic expression of 67 newly diagnosed WHO grade III and IV gliomas using DNA microarray and correlated the gene expression with prognosis. They found that the pro‐neural subtype expresses OLIG2, DLL3, and BCAN and is more prevalent in younger patients with better prognosis.^[^ ^22^ ^]^ On the other hand, the mesenchymal subtype (YKL40, CD44, and VEGF overexpression) and proliferative subtype (PCNA and TOP2A enriched) showed poor prognosis and were primarily associated with older patients.^[^ ^22^ ^]^

**Figure 1:**
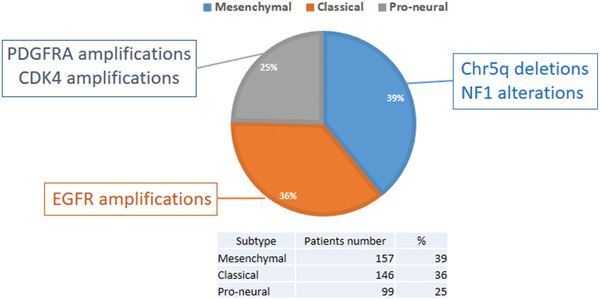
TCGA analysis for distribution of GBM subtypes at diagnosis and their associated markers.

Later, a combined genetic analysis collected from three different sources by The Cancer Genomic Atlas (TCGA) identified 840 genes and classified GBM into four different subtypes: pro‐neural, neural, classical, and mesenchymal.^[^ ^12^ ^]^ The classical subtypes are characterized by lack of TP53 mutation, EGFR amplification associated with expression of retinoblastoma (RB), sonic hedgehog, and Notch signaling pathway. The mesenchymal subtypes are characterized by NF1 and PTEN comutation along with Akt and NF‐kb signaling pathway enrichment. Of note, the Akt signaling pathway enrichment in MES phenotype was also identified in the study of Phillips et al.^[^ ^22^ ^]^ PDGFRA and TP53 aberration, and IDH1 mutation were associated with pro‐neural subtypes. The neural subtypes mainly express neuronal markers (NEFL, GABRA1, SYT1, and SLC12A5), but later studies showed that this subtype is simply a contamination with nontumoral cells.^[^ ^23^, ^24^, ^25^ ^]^ **Figure** **1** summarizes the genetic alterations/amplifications of each subtype and the relative rate of each subtype at diagnosis (per TCGA analysis). Both Phillips et al. and Verhaak et al. demonstrated that patients with the pro‐neural GBM subtype are younger in age and tend to survive longer.^[^ ^12^, ^22^ ^]^ But, another study conducted by Sturm et al. concluded that, the favorable outcome of PN patients is due to IDH mutation.^[^ ^23^ ^]^

In 2017, TCGA further identified three subtypes of GBM, namely, classical, pro‐neural, and mesenchymal where they correlated intrinsic gene signature with tumor immune microenvironment.^[^ ^14^ ^]^ In this study, they recruited patients with no IDH mutation as they showed better prognosis in previous studies^[^ ^23^ ^]^ and established the relationship between treatment and phenotypic alterations with GBM subtypes in recurrent tumors. They identified 150 genes and nearly 50% of them overlapped with their previous classification based on 840 genes. Interestingly, different genetic analysis platform (RNA‐seq and Affymetrix‐U133A) and multiple samples from the same tumor showed concordance among subtypes. In an effort to measure phenotypic plasticity upon recurrence, they compared 91 recurrent with primary gliomas carrying wild type IDH and found that the PN subtype had the highest plasticity rate with 59% of cases shifting to other subtypes, compared to 35% of MES subtype.^[^ ^14^ ^]^

The last robust study conducted by Neftel et al. classified 28 IDH‐wild type GBMs (including adult and children glioblastomas) into six metamodules based on extensive gene expression analysis, correlating GBM subtypes with cycling states and plasticity, shedding light on intratumoral heterogeneity.^[^ ^26^ ^]^ Among these modules, two metamodules predominantly expressed mesenchymal‐like genes (e.g., vimentin, termed mesenchymal like), where one of them highly expressed hypoxia‐response genes, stress, and glycolytic genes indicating that hypoxia and increased glycolysis may involve in MES‐like heterogeneity, also supported by other studies.^[^ ^27^, ^28^ ^]^ Metamodule 3 primarily expressed astrocytic markers (S100B, GFAP, SLC1A3, GLAST, and MLC1) termed as astrocytic (AC‐like), whereas metamodules 4 was termed oligodendrocytic precursor cells (OPC‐like) as they expressed oligodendrocytic lineage markers (OLIG1, OMG, PLP1, PLLP, TNR, and ALCAM). Neural progenitor cell (NPC) markers (e.g., SOX4, SOX11, and DCX) are enriched in metamodules 5 and 6, naming them as NPC‐like subtypes. Overall, they identified four cellular states in GBM, MES‐like, AC‐like, OPC‐like, and NPC‐like and observe that the same tumor contains at least two cellular states (most of them had four states), but particular genetic expression defined their cellular states in most cases. By comparing the bulk genetic expression of 401 samples from TCGA dataset, they identified that EGFR amplification is associated with AC‐like metamodules, whereas point mutation of NF1 is associated with MES‐like clusters consistent with Verhaak et al.^[^ ^12^ ^]^ Similarly, PDGFRA and CDK4 amplification are correlated with OPC‐like and NPC‐like scores, respectively, in line with previous work.^[^ ^29^, ^30^ ^]^ In an attempt to compare their results with TCGA classification, they compare fraction of cells in four cellular states in each tumor with TCGA cohort. Ultimately, they noticed that the AC‐like and MES‐like metamodules correspond to TCGA classic and mesenchymal subtypes, respectively, whereas OPC‐like and NPC‐like together coincide with TCGA pro‐neural subtype.

## 3 Molecular Mechanisms of Mesenchymal Transformation in GBM

It is now well known that GBMs are dynamic tumors that tend to have phenotypic plasticity upon recurrence and in response to radio/chemotherapy, leading to resistance. Wang et al. reported that over 30% of primary GBMs change their subtypes upon recurrence,^[^ ^31^ ^]^ and this cellular plasticity is common in GBM with wild‐type IDH.^[^ ^14^ ^]^ A recent study confirmed this idea^[^ ^26^ ^]^ by barcoding a specific subtype before orthotopic implantation in mice followed by single cell RNA sequencing (RNAseq) and barcode analysis of different GBM state in the same tumor. This phenotypic plasticity is believed to be the main driver of GBM heterogeneity. Although the accurate classification of GBM subtypes varies in the literature, the PN and MES subtypes are consistently identified and appear to be robust among different classification systems. PN subtype patients tend to be younger and tend to survive longer compared to patients with MES tumors.^[^ ^14^ ^]^ Several studies demonstrated that a single cell subtype can contribute to different GBM states^[^ ^26^ ^]^ and other subtypes may arise from PN tumors.^[^ ^22^, ^32^ ^]^ This observation supports the idea that, unidirectional shift from pro‐neural to mesenchymal may happen in response to treatment and/or tumor recurrence. Phillips and colleagues demonstrated that PN tumor acquired mesenchymal characteristics during recurrence,^[^ ^22^ ^]^ losing OLIG2 expression (a PN‐specific marker) while gaining the mesenchymal marker YKL40, and termed this cellular plasticity as PMT, similar to EMT in solid tumors of epithelial origin. Other studies supported this PMT in GBM^[^ ^33^, ^34^, ^35^ ^]^ and showed that tafazzin (TAZ), signal transducer and activator of transcription 3 (STAT3), and CCAAT enhancer binding protein beta (C/EBP‐*β*) are responsible for reprograming PN glioma stem‐like cells (GSCs) into MES subtype, while the nuclear factor kappa‐light‐chain‐enhancer of activated B cells (NF‐kB) act as the main mediator. Multiple factors, such as genetic, epigenetic, and tumor microenvironment (TME), could act as the driving force for GBM phenotypic plasticity. In the following section, we will discuss the specific factors (molecules, transcription factors, pathways, and tumor microenvironment) involved in mesenchymal transformation, summarized in **Table** **1**.

**Table 1:** The regulatory molecules/pathways involved in MES transition of GBM

	Role in PMT	Target(s)	Impacts on GBM progression	References
Extracellular stimuli				
TGF‐*β*	Inducer	Smad2, PDK1/c‐JUN, NF‐*κ*B	Enhance migration, invasion, proliferation, tumor growth, and metastasis	^[^ ^37^, ^38^, ^39^, ^40^, ^41^, ^42^, ^43^, ^44^, ^45^, ^46^, ^47^ ^]^
TNF‐*α*	Inducer	NF‐*κ*B		
IL‐8	Inducer	NF‐*κ*B		
Wnt	Inducer	*β*‐catenin		
Signaling molecules				
C/EBP‐*β*	Inducer	DNA sequence	Attenuate proliferation, migration, invasion, and mesenchymal properties	^[^ ^34^, ^35^, ^52^, ^53^, ^54^, ^55^, ^142^, ^143^ ^]^
STAT3	inducer	DNA sequence		
ZEB1	Inducer	DNA sequence		
TWIST1	Inducer	DNA sequence		
FOXD1	Inducer	DNA sequence		
SNAI1	Inducer	DNA sequence		
Epigenetic control				
EZH2	Inducer	TGIF2	Modulate the mesenchymal properties with enhanced aggressiveness	^[^ ^65^, ^66^, ^67^, ^68^, ^71^ ^]^
MeCP2	Inducer	DNA sequence		
ZBTB18	Inducer	DNA sequence		
microRNAs				
miR‐205	Suppressor	ZEB1	Suppress migration, invasion, stemness, and mesenchymal phenotype	^[^ ^47^, ^75^, ^76^, ^77^, ^78^, ^80^, ^81^, ^144^ ^]^
miR‐590‐3p	Suppressor	ZEB1, ZEB2		
miR‐504	Suppressor	FZD7		
miR‐140	Suppressor	CTSB		
miR‐101‐3p	Suppressor	TRIM44		
miR‐517c	Suppressor	KPNA4		
miR‐96	Suppressor	AEG1		
miR‐211	Suppressor	HMGA2		
miR‐10b	Inducer	Apaf‐1, PTEN, E‐cad	Enhance proliferation, migration, and invasion	^[^ ^82^ ^]^
Long noncoding RNAs				
LINC00511	Inducer	YB1	Increase proliferation, invasion, migration, and MES phenotype	^[^ ^83^, ^84^, ^85^ ^]^
LINC00645	Inducer	ZEB1		
TALNEC2	Inducer	miR‐21, miR‐91		
LINC00599	Suppressor	MES genes	Inhibit migration, invasion, and mesenchymal transition	^[^ ^86^ ^]^

### 3.1 Extracellular Stimuli

**Figure 2:**
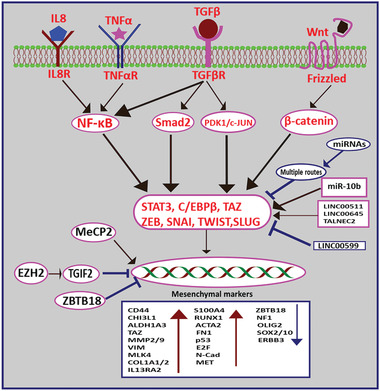
The regulatory mechanisms involved in GBM MES transformation . Extracellular soluble factors (such as interleukin 8 (IL8), tumor necrosis factor‐*α* (TNF*α*), transforming growth factor beta (TGF*β*), and Wnt) bind to their respective receptors on GBM cells and induce MES transition by initiating a cascade of molecular events. Epigenetic reprogramming [enhancer of zeste homolog 2 (EZH2), zinc finger, and BTB domain containing 18 (ZBTB18)] modulate MES transition by direct binding to DNA molecules. MicroRNAs (miRNAs) and long noncoding RNAs (LNC) can also contribute to MES transition by modulating their target. Intermediate molecules: nuclear factor kappa light chain enhancer of activated B cells (NF‐*κ*B), Smad family member 2 (Smad2), pyruvate dehydrogenase kinase 1 (PDK1)/proto‐oncogene c‐JUN (c‐JUN); Transcription factors: tafazzin (TAZ), signal transducer and activator of transcription 3 (STAT3) and CCAAT enhancer binding protein beta (C/EBP‐*β*), zinc finger protein SNAI (SNAI), zinc finger protein SNAI2 (SLUG), zinc finger E‐box‐binding homeobox (ZEB), twist‐related protein (TWIST).

External stimuli from the TME play a significant role in GBM mesenchymal transition. Accumulating evidence suggest that the transforming growth factor‐*β* (TGF‐*β*) is an important player. Exposure of GBM cells to TGF‐*β* upregulated fibronectin and COL5A1 mesenchymal markers, leading to an aggressive behavior characterized by increased neurosphere formation and spindle‐shaped morphology and a more scattered growth pattern and this effect was reversed with TGF‐*β* inhibition.^[^ ^36^ ^]^ Mechanistically, TGF‐*β* induces ZEB1 activity through SMAD2 phosphorylation, causing increased aggressiveness/invasiveness.^[^ ^36^ ^]^ Canonical TGF‐*β* dependent mesenchymal transition is also confirmed by other studies. Wang and colleagues demonstrated that Smad‐dependent BACE2 induction by TGF‐*β* modulates MES transition through tumor necrosis factor alpha (TNF‐*α*)‐induced NF‐*κ*B activation and PP1A/IKK pathway.^[^ ^37^ ^]^ Noticeably, TGF‐*β* expression in necrotic areas is associated with TWIST expression, a strong mesenchymal marker.^[^ ^38^ ^]^ In addition to canonical pathway, TGF‐*β* induces noncanonical pathway which could also be involved in MES transformation. Luo et al. concluded that TGF‐*β* can induce MES transformation through PDK1/c‐Jun pathway by activating N‐cadherin, ZEB1, SNAIL, and TWIST1.^[^ ^39^ ^]^ Consistently, in neuroblastoma, TGF‐*β* can induce GLI expression, modulating mesenchymal transition through Smad‐independent pathway.^[^ ^40^ ^]^ Mesenchymal transition by IL‐8 has also been demonstrated in GBM^[^ ^41^ ^]^ and elevated level of this chemokine is found in recurrent GBMs.^[^ ^42^ ^]^ IL‐8 expression is also higher in paired GBM tissue samples, in comparison to adjacent nontumor tissue from the same patient^[^ ^41^ ^]^ and in GBM cell lines.^[^ ^41^, ^43^, ^44^ ^]^ In an attempt to identify the role of IL‐8 in GBM mesenchymal transformation, Zhang et al. measured N‐cadherin expression in IL‐8 overexpressed cells and found upregulated levels which corresponded with vimentin expression, however, knockdown experiment showed the opposite trend.^[^ ^41^ ^]^ They suggested that the underline MES transition and aggressiveness may occur through ELMO1‐NF‐*κ*B‐Snail signaling cascade in an autocrine fashion.^[^ ^41^ ^]^ In another study, Conroy et al. found that elevated levels of IL‐8 are associated with MES subtype, but the master regulators of MES transition (STAT3, C/EBP‐*β*, and TAZ) were not changed upon treatment of PN subtype with IL‐8, suggesting that IL‐8 may control MES transition by paracrine fashion^[^ ^45^ ^]^ consistent with Raychaudhuri's findings.^[^ ^44^ ^]^ Further studies revealed that MES subtype secreted IL‐8 act as a proangiogenic factor, affecting endothelial cells (ECs) properties in GBM microenvironment in a paracrine fashion, ultimately inducing PMT.^[^ ^45^ ^]^ Another extracellular stimuli known as Wnt was demonstrated to be a mesenchymal inducer in GBM. FZD6 (a negative regulator of Wnt signaling) overexpression increased GBM aggressiveness, decreasing PN marker (OLIG2 and SOX2) and increasing MES genes (ALDH1A3 and CD44).^[^ ^46^ ^]^ Consistently, knockdown and inhibition of Wnt by pharmacological agents confirmed its association with PMT.^[^ ^46^ ^]^ A similar effect of FZD7 on mesenchymal transformation was also demonstrated in different GBM cell lines.^[^ ^47^ ^]^ The major signaling pathways associated with MES transition are summarized in **Figure** **2**.

In response to external stimuli induced by the tumor cell intrinsic and TME factors, NF‐*κ*B is activated leading to mesenchymal transformation in various cancer types including GBM. One of the most reported external stimuli associated with GBM MES transition via NF‐*κ*B is TNF‐*α*. Treatment of PN GSCs with TNF‐*α* causes MES shift characterized by high expression of CD44^[^ ^35^ ^]^ and addition of I*κ*B‐SR (an NF‐*κ*B inhibitor) can reverse this activity, supporting the relationship between TNF‐*α* and NF‐*κ*B in PMT.^[^ ^35^ ^]^ Iwata et al. reported that TNF‐*α* treatment of PN GSCs dramatically increased NF‐*κ*B activity, while silencing of NF‐*κ*B attenuated ICOSLG expression (typically associated with MES) after TNF‐*α* treatment.^[^ ^48^ ^]^ Another interesting work relates to the involvement of serine/threonine kinase MLK4 in TNF‐*α* mediated NF‐*κ*B activation and mesenchymal transformation.^[^ ^49^ ^]^ Two other review articles cover the other aspects of the NF‐*κ*B role in mesenchymal transformation and we will not further discuss here.^[^ ^17^, ^50^ ^]^

### 3.2 Core Transcriptional Regulators

Transcriptional factors share similar cellular hierarchies in controlling GBM tumorigenesis and mesenchymal transformation. In order to identify the regulatory mechanism of GBM MES transformation, Iavarone and co‐workers performed comprehensive integrative genomic analysis and concluded that two transcription factors (C/EBP‐*β* and STAT3) act as the master regulators in MES transition.^[^ ^33^ ^]^ C/EBP‐*β* expression was found to be upregulated in GSCs upon radiation and/or in response to TNF‐*α* treatment^[^ ^51^ ^]^ along with CD109 (MES‐specific marker), indicating that this transcription factor is involved in PMT. Berendsen et al. demonstrated that the subventricular zone of the brain can modulate GBM microenvironment and take part in MES transformation through C/EBP‐*β* signaling.^[^ ^52^ ^]^ Another study reported that transglutaminase 2 (TGM2) can act as a mediator of mesenchymal transformation in perinecrotic regions of GBM by inducing C/EBP‐*β* expression through GADD153 (a transcriptional inhibitor of C/EBP*β* signaling) degradation.^[^ ^53^ ^]^ Accumulating evidence demonstrated the role of STAT3 in PMT. Chesnelong et al. reported that the progenitor‐like GSCs with mesenchymal features arise in a STAT3‐dependent manner.^[^ ^54^ ^]^ Mechanistically, STAT3 can modulate the activity of SLUG, inducing mesenchymal transformation, while progenitor‐like GSCs acquire more aggressive phenotype.^[^ ^54^ ^]^ In line with this work, STAT3/SLUG axis can induce mesenchymal transformation in irradiated/invasive patient‐derived GSCs.^[^ ^55^ ^]^ Moreover, addition of AZD1480 (a STAT3 inhibitor) to radiation therapy leads to an effective reduction of tumor burden in mice PN xenograft models.^[^ ^56^ ^]^ These data were further confirmed with another STAT3 inhibitor, HJC0152, on patient‐derived cells.^[^ ^57^ ^]^ The role of TAZ and Hippo signaling pathway, along with other molecules, in mesenchymal transformation were recently described by Fedele et al.^[^ ^17^ ^]^

### 3.3 Epigenetic Regulation

Epigenetic regulation by histone modification, DNA methylation, and chromatin remodeling plays a critical role in pathological conditions, for instance, tumor initiation, progression, and EMT.^[^ ^58^, ^59^, ^60^ ^]^ Malignant GBM transformation by aberrant histone alteration is evident in recent literature.^[^ ^61^, ^62^, ^63^ ^]^ In particular, abnormal histone modification induces inactivation of cancer repressor genes, leading to abnormal GBM growth. The histone‐lysine N‐methyltransferase enzyme EZH2 (enhancer of zeste homolog 2), which catalyzes the addition of methyl groups to histone H3 at lysine 27 thereby silencing gene function, plays an important role in glioma progression.^[^ ^64^, ^65^ ^]^ EZH2 inhibitor GSK343 upregulates EZH2 target genes, leading to suppression of GBM growth and reversing PMT by controlling N‐cadherin and vimentin expression.^[^ ^66^ ^]^ Vinchure et al. demonstrated that epigenetic control of miR‐490‐3p by EZH2 regulates PMT through TGIF2 signaling.^[^ ^67^ ^]^ The methyl CpG‐binding protein 2 (MeCP2) induces PMT through miR‐200 family, which in turn targets ZEB1 and ZEB2 expression through epigenetic modification.^[^ ^68^ ^]^ DNA hypo‐ and hypermethylation of critical genes is also associated with malignant transformation of brain tumors.^[^ ^69^, ^70^ ^]^ Accumulating evidence suggests that PMT regulation by epigenetic controlled DNA methylation is associated with glioma progression.^[^ ^71^ ^]^ Zinc finger and BTB domain containing 18 (ZBTB18) gene is a transcriptional tumor suppressor associated with brain development and neuronal differentiation.^[^ ^72^, ^73^ ^]^ A study using TCGA high‐ and low‐grade glioma gene signature analysis showed that high frequency of ZBTB18 gene hypermethylation occurred primarily in MES subtype (compared to PN) .^[^ ^71^ ^]^ Ectopic expression of ZBTB18 in patient‐derived GBM cells is associated with higher mesenchymal markers and overexpression study showed the opposite trend.^[^ ^71^ ^]^ Moreover, an inverse association of ZBTB18 promoter methylation with selected MES genes (CD97, ACTN1, EMP3, and CHI3L1) confirms its role in PMT epigenetic regulation. Thus, one can conclude that epigenetic alteration is prevalent in GBM pathogenesis and regulatory mechanisms can cooperate and co‐opt with PMT in this tumor.

A group of small noncoding RNAs called microRNAs (miRNAs) are known to associate with cancer cell proliferation, migration, invasion, differentiation, metastasis, and survival. The role of miRNAs in PMT is also established. Zhang et al. analyzed miRNAs and mRNAs profile of 491 TCGA dataset and identified 18 miRNAs to positively correlate with PMT associated genes, while 19 miRNAs showed the opposite effect.^[^ ^74^ ^]^ Among those miRNAs, overexpression of miR‐95 negatively correlated with PMT (marked by expression of N‐catenin) while miR‐223 showed the opposite trend. Both miR‐205 and miR‐590‐3p associated with more epithelial‐like characteristics, targeting ZEB transcription factor.^[^ ^75^, ^76^ ^]^ Clinically, expression levels of both miRNAs were attenuated in GBM tissues and cell lines compared to non‐neoplastic samples. Mechanistically, miR‐205 targets 3′‐UTR region of ZEB1 and induces the expression of E‐cadherin, while suppressing N‐cadherin and vimentin.^[^ ^75^ ^]^ A study using transcription data from TCGA showed that miR‐504 downregulation is associated with GBM MES subtype and poor survival, while overexpression of this miRNA suppresses mesenchymal transition and tumor aggressiveness.^[^ ^47^ ^]^ miR‐504 targets the 3′‐UTR region of FZD7 and modulate the activity of Wnt‐*β*‐catenin signaling. Silencing and overexpression studies confirmed the role of miR‐504 in GBM stemness, another closely related phenomenon in PMT. Similarly, miR‐140, miR‐101‐3p, miR‐577, miR‐96, and miR211 suppressed GBM mesenchymal properties by targeting cathepsin B, TRIM44, Rab25, AEG‐1, and HMGA2, respectively.^[^ ^77^, ^78^, ^79^, ^80^, ^81^ ^]^ On the other hand, aberrant expression of miR‐10b is associated with grade IV gliomas as well as PMT.^[^ ^82^ ^]^ Agomir (a miRNA mimic) of miR‐10b injection enhances tumor growth in vivo, while miR‐10b mimics induces a significant morphological change of GBM cells from pebble shape to the long fusiform shape, a feature of PMT.^[^ ^82^ ^]^

Long noncoding RNAs (lncRNAs) have also been shown to play a pivotal role in GBM pathogenesis and mesenchymal transformation. For example, the long intergenic noncoding RNA 00511 (LINC00511) is upregulated in GBM tissues, and its overexpression in GBM cells leads to induction of mesenchymal markers, while downregulating E‐cadherin levels. Mechanistically, ZEB1 regulates LINC00511 activity at the transcription level and ultimately control PMT through LINC00511/miR‐524‐5p/YB1/ZEB1 positive feedback loop.^[^ ^83^ ^]^ LINC00645 is another lncRNA upregulated in GBM and shown to regulate PMT through ZEB1, acting through LINC00645/miR‐205‐3p/ZEB1 signaling axis, potentially triggered by TGF‐*β*.^[^ ^84^ ^]^ TALNEC2 was also found to be increased in GBM and associated with PMT .^[^ ^85^ ^]^ Downregulation of TALNEC2 in GSCs affects the expression of PMT‐associated genes.^[^ ^85^ ^]^ In contrast to the positive relationship between lncRNAs and GBM, Fu et al. suggested that LINC00599 acts as a tumor suppressor, where upregulation of this lncRNA increases E‐cadherin expression, while decreases vimentin in GBM cells.^[^ ^86^ ^]^

### 3.4 The Tumor (Immune) Microenvironment

**Figure 3:**
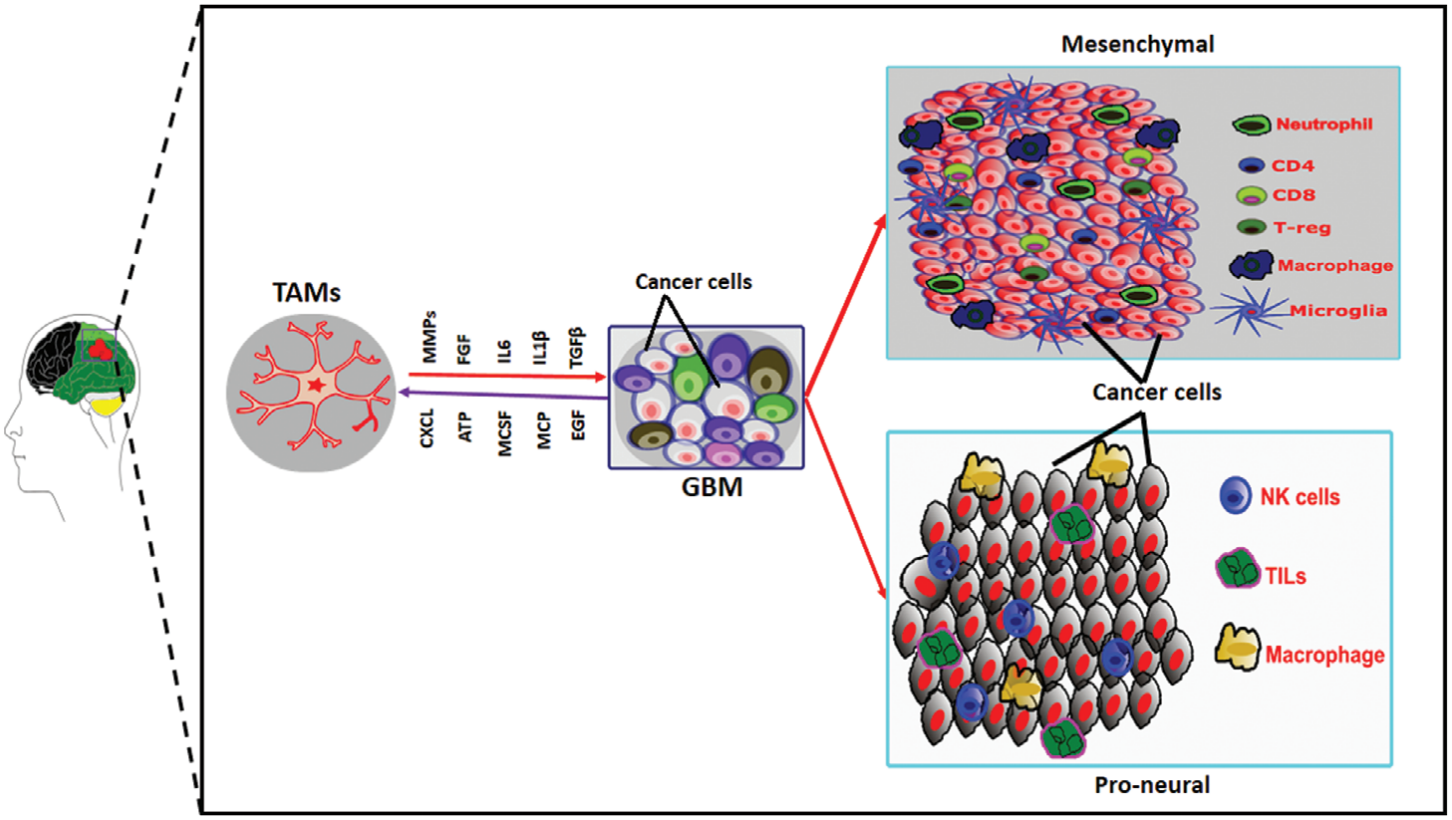
Immune microenvironment triggers MES signature in GBM. A bilateral interaction between GBM and tumor associated macrophages/microglia (TAMs) through soluble factors and other regulatory mechanisms can induce mesenchymal transformation. MES tumors are characterized by the presence of different types of T‐cells, neutrophils, macrophages, and microglia. On the other hand, natural killer (NK) cells and tumor‐infiltrating lymphocytes (TILs) are predominant in PN tumors.

Tumor‐associated microglia/macrophages (TAMs) are considered the largest stromal cell populations in GBM TME and thought to also be involved in PMT.^[^ ^14^, ^87^ ^]^ Single cell RNAseq revealed that nearly 50% of cells in GBMs TME are infiltrative immune cells, of which 95% are resident microglia/macrophages and the rest are mainly dendritic cells.^[^ ^88^ ^]^ Similar observation by Wang and colleagues showed enriched infiltrative lymphocytes in the MES subtype.^[^ ^89^ ^]^ Subtype‐specific immune profile also demonstrated that GBM MES phenotype is enriched with pro‐inflammatory and immunosuppressive genes (summarized in **Figure** **3**).^[^ ^90^, ^91^, ^92^, ^93^ ^]^ In line with previous work, two recent studies demonstrated that TAMs are mainly enriched with GBM MES subtype. Neftel and colleagues found that the preponderance of TAMs is higher in MES tumors, while Kaffes et al. showed that the number of immune cells are proportionally associated with this subtype.^[^ ^26^, ^94^ ^]^ Further, infiltration of T cell populations of all classes (T helper, cytotoxic, and regulatory) is higher in MES GBM, compared to PN and CL.^[^ ^94^ ^]^ These findings are also supported by Beier and colleagues where they showed that CD8^+^ T cells are mainly associated with MES tumors.^[^ ^92^ ^]^ In another study, 98 newly diagnosed GBM tissues were analyzed by immunohistochemistry which showed that MES tumors were enriched with microglia and macrophage infiltration and higher CD4+ cells,^[^ ^95^ ^]^ with no difference in cytotoxic T lymphocytes, inconsistent with other studies.^[^ ^92^, ^94^ ^]^ More than 40% of TCGA dataset MES phenotype are enriched with tumor‐infiltrating lymphocytes but not with activated natural killer cells.^[^ ^14^ ^]^ Presence of T lymphocytes in MES GBM was further solidified by other studies, showing that knockdown of NF1 enhances macrophage recruitment^[^ ^14^ ^]^ as NF1 deletion/alteration is common in MES subtype.^[^ ^14^, ^26^ ^]^ Moreover, Rutledge et al. demonstrated that NF1 mutation along with absence of EGFR amplification and PTEN deletion are strongly associated with T lymphocytes in GBM.^[^ ^96^ ^]^ The relationship between T lymphocytes and necrosis (characteristic feature of MES subtype) remains controversial; Cooper et al. showed that MES tumor with higher T lymphocytes did not have higher necrosis, suggesting their role in adaptive immunity but not in necrosis.^[^ ^97^ ^]^ Although the interaction between TAMs and GBMs depends on multiple factors (e.g., hypoxia), GBM‐derived secretome (chemokines, cytokines, and soluble factors) can activate and recruit TAMs in the tumor niche.^[^ ^98^ ^]^ Once actively recruited to GBM, TAMs release VEGF and CXC‐chemokine ligand 2 (CXCL2) in the TME necessary for neovascularization.^[^ ^99^ ^]^ GBM‐derived colony stimulating factor 1 (CSF1) can activate microglia, which in turn can induce angiogenesis through insulin‐like growth factor‐binding protein 1 (IGFBP1) secretion.^[^ ^100^ ^]^ GBM‐associated vascular ECs can also transform to a MES phenotype, increasing their ability to proliferate and migrate, ultimately promoting tumorigenesis and chemoresitance.^[^ ^101^, ^102^, ^103^ ^]^ Aberrant vasculature in GBM microenvironment was observed when ECs acquired MES phenotype through c‐Met signaling.^[^ ^102^ ^]^ Mechanistically, c‐Met can activate ETS‐1/MMP‐14 leading to vascular malfunction.^[^ ^102^ ^]^ Another study demonstrated that ECs acquired mesenchymal stem‐like cell characteristics through c‐Met/*β*‐catenin/MRP‐1‐dependent mechanism, conferring chemoresistance in GBM.^[^ ^103^ ^]^ Moreover, MES transformation of ECs can be induced by NF‐*κ*B‐dependent Snail expression under platelet‐derived growth factor (PDGF) induction, while pharmacological and genetic inhibition of PDGF signaling sensitizes GBM to anti‐VEGF/VEGFR therapy.^[^ ^101^ ^]^

Hypoxia, a characteristic feature of solid tumors including GBM, can also induce mesenchymal transformation by regulating hypoxia‐inducible factors (HIF) and proteins, controlling a vast array of gene expression related to mesenchymal transformation in various cancers.^[^ ^104^ ^]^ Joseph et al. demonstrated that the hypoxia‐dependent mesenchymal transformation is directly controlled by HIF1*α* (but not HIF2*α*), by regulating ZEB1 expression in GBM, and detected hypoxia marker (GLUT1) and mesenchymal markers (ZEB1 and YKL40) in patient‐derived tissues.^[^ ^27^ ^]^ Knockdown or pharmacological inhibition of HIF1*α* can effectively reverse hypoxia‐regulated MES transformation. In contrast, Qiu et al. reported that hypoxia‐mediated mesenchymal transformation occurred through HIF2*α*, indicating the refractory nature of GBM.^[^ ^105^ ^]^ Mechanistically, HIF2*α* stabilizes the tyrosine kinase receptor EPHB2 under hypoxic conditions, ultimately controlling MES transformation by phosphorylating paxillin and focal adhesion kinase (FAK).^[^ ^105^ ^]^ Hypoxia can also modulate the production of certain metabolite in extracellular GBM TME such as excess adenosine production, enhancing MES transformation through HIF‐2/PAP‐dependent activation of A3AR.^[^ ^106^ ^]^ Hypoxia can modulate MES transition through other routes such as macrophage migrating inhibiting factor (MIF), through MIF‐CXCR4‐AKT regulatory pathway.^[^ ^107^ ^]^

The crosstalk between immune cells and mesenchymal transition is a two‐way interaction probably mediated by TAMs. TAMs secrete extracellular matrix remodeling factors along with proangiogenic anti‐inflammatory cytokines which together change the tumor properties toward a more aggressive phenotype. Wang et al. found that recurrent GBMs with mesenchymal transition are associated with M2 macrophage (a state of macrophage polarization believed to support tumor growth), and the same TAMs can exert their effect on tumors enhancing MES phenotype.^[^ ^14^ ^]^ In summary, all evidence strongly suggest that the bilateral interaction between immune microenvironment and GBMs can induce mesenchymal transformation.

## 4 Therapy Resistance and Clinical Implications of Mesenchymal Transformation in GBM

In addition to maximal resection of the tumor (considered as a first line treatment for GBM), additional treatment modalities consist of ionizing radiation, alkylating chemotherapy (Temozolomide, TMZ), and potentially anti‐angiogenic agents (anti‐VEGF). However, in terms of efficacy, these treatments are still considered ineffective as the progression‐free survival and overall survival remains to be poor. After initial diagnosis, more than 90% of GBM patients die within five years.^[^ ^108^ ^]^ All of these treatment modalities might impact the intrinsic nature of GBM through mesenchymal transition, ultimately affecting patients survival.^[^ ^109^, ^110^, ^111^ ^]^

Both primary and recurrent GBM with mesenchymal signature tend to show worst prognosis compared to the PN subtype.^[^ ^18^ ^]^ The glioma stem cell subpopulation, believed to initiate the tumor, can largely adapt to conventional therapy and eventually develop resistance.^[^ ^112^ ^]^ Marziali et al. showed that GSCs can be divided into two main classes which share either MES or PN gene signature.^[^ ^113^ ^]^ Radiation treatment of PN GSCs induces PMT through CD44 overexpression and NF‐kB activation, leading to radioresistance.^[^ ^35^ ^]^ Segerman and colleagues demonstrated that clones resistant to multitherapy (both radiation and multiple drugs) tend to show mesenchymal‐like characteristics, while clones sensitive to therapy are of PN‐like phenotype.^[^ ^16^, ^111^ ^]^ TMZ targets only proliferating cells (not GSCs)^[^ ^114^ ^]^ and patients with methylated MGMT promoter show better response to TMZ.^[^ ^115^ ^]^ To elucidate the relationship between TMZ resistance and PMT, Wang and colleagues established TMZ‐resistant cell lines (by exposing cells to TMZ for six months) and observed that mesenchymal markers increased as resistance developed.^[^ ^18^ ^]^ Moreover, TMZ‐resistant cells showed phenotypic changes (increased invasion, attachment, and detachment) related to MES phenotype. More recently, Tejero et al. showed that a small group of quiescent cells in GBM cultures are more aggressive and therapy resistance.^[^ ^116^ ^]^ By gene set enrichment analysis (GSEA), they identified that most of the PMT‐associated genes were upregulated in therapy‐resistant quiescent cells.^[^ ^116^ ^]^ TMZ intrinsic and/or acquired resistance in GBM is not regulated by a single molecular event, rather by multiple proteins and TFs acting together. Recent study suggested that the forkhead box protein O1 (FOXO1) plays a crucial role in TMZ resistance and MES transformation.^[^ ^117^ ^]^ Other studies demonstrated that the histone 2A family member J (H2AFJ) is involved in TMZ resistance and MES transformation through NF‐*κ*B and STAT3‐dependent signaling.^[^ ^118^, ^119^ ^]^ GSCs can induce an immunosuppressive nature in TAM through mTOR‐dependent regulation of STAT3 and NF‐*κ*B activity.^[^ ^120^ ^]^ Our group recently reported that GSCs with MES subtype can force PN cells to switch into a MES phenotype by means of extracellular vesicles (EVs),^[^ ^121^ ^]^ a small vesicle released by practically every cell.^[^ ^122^ ^]^ We showed that PN GSCs can uptake MES‐derived EVs contributing greatly to GBM intratumoral heterogeneity and therapeutic resistance through NF‐*κ*B/STAT3‐dependent manner.^[^ ^121^ ^]^ In another study, macrophage‐derived small EVs were shown to induce PMT by targeting CHD7 in PN GSCs.^[^ ^123^ ^]^

As GBMs are highly angiogenic, it is believed that anti‐angiogenic therapy (bevacizumab) may reduce the disease burden, but practically, this therapy induces mesenchymal transformation ultimately leading to therapy failure. Huang et al. showed that bevacizumab induces PMT in glioma cells characterized by decreased E‐cadherin along with increased N‐cadherin and vimentin.^[^ ^109^ ^]^ Piao and colleagues performed gene expression analysis of bevacizumab‐resistant cells and found that these cells are associated with mesenchymal‐like characteristics^[^ ^110^ ^]^ along with increased invasion, migration, and pro‐inflammatory cytokines expression. Chandra et al. reported that bevacizumab‐resistant GBMs are enriched with mesenchymal signature with increased stemness along with altered morphology and perivascular invasiveness.^[^ ^124^ ^]^ Single‐cell analysis and CRISPR‐Cas9‐mediated gene knockout in paired patients of bevacizumab‐resistant GBMs and PDX models revealed that ZEB1 drives this MES transformation by altering the metabolic state of bevacizumab‐resistant cells through GLUT3 activity.^[^ ^124^ ^]^ Another study demonstrated that GBM initially responds to bevacizumab, but in most cases the tumor recur, concluding that targeting MET (a PMT regulator) and VEGF simultaneously may be a more effective therapy.^[^ ^125^ ^]^ Other studies suggest that bevacizumab‐induced tumor progression related to MES phenotype is mediated through MET in a hypoxia‐dependent manner.^[^ ^110^, ^126^ ^]^ Surgery may also act as a confounding factor in PMT, as it has been shown that injured tissue may trigger wound healing process in other cancer.^[^ ^127^ ^]^

Both clinical and preclinical studies demonstrated that patients with PN GBM subtype respond better to radio and chemotherapy, with a better prognosis compared to patients with the MES subtype.^[^ ^14^, ^16^, ^35^ ^]^ IDH mutations are common in the PN subtype, thus responsible for the favorable outcome in this subtype.^[^ ^23^ ^]^ Brennan and others^[^ ^128^, ^129^ ^]^ demonstrated that PN GBMs manifest the cytosine‐phosphate‐guanine island methylator phenotype (G‐CIMP) and tumors with both IDH1 mutation and G‐CIMP have better prognosis, while G‐CIMP negative and wt IDH1 GBM show similar prognosis to the MES subtype. Thus, subtype‐specific mutations can highlight therapy resistance in GBM. PN subtype patients tend to be younger^[^ ^115^ ^]^ and some studies suggested that other GBM subtype may arise from PN cells.^[^ ^32^ ^]^ Thus, one could speculate that a constant phenotypic shifting occurs, and ultimately PN cells turn into MES subtype and confer therapeutic resistance.^[^ ^16^, ^111^ ^]^ Herting et al. developed high grade glioma mouse model with NF1 silencing and PDGFB overexpression, corresponding to MES and PN phenotype, respectively. PDGFB overexpressing PN model showed higher sensitivity to radio and TMZ therapy with preference to radio therapy over TMZ, whereas NF1‐deleted MES model demonstrated less sensitivity to both conventional therapies.^[^ ^130^ ^]^ Interestingly, a recent finding suggests that the diacylglycerol kinase alpha (DGK*α*) inhibitor targets GBMs in which PMT has occurred.^[^ ^131^ ^]^ These results are in line with other studies showing that the MES subtype is more sensitive to the epigenetic regulator BMI.^[^ ^132^ ^]^ In both cases, they act synergistically with conventional radio and chemotherapy.^[^ ^131^, ^132^ ^]^ In addition to therapy selection, PMT studies may be helpful in diagnostic imaging. Magnetic resonance spectroscopy study successfully identified elevated 2 hydroxyglutarate and hyperpolarized [1‐(13)C] lactate level in IDH 1 mutant GBM without biopsy.^[^ ^133^, ^134^ ^]^

## 5 Tools to Study Mesenchymal Transformation

GSC cultures derived from patient tissues with different subtypes are considered useful and clinically relevant tools to study mesenchymal transformation, typically measured by simple molecular biology techniques.^[^ ^18^, ^35^ ^]^ For example, Bhat et al. treated a set of GSCs with TNF‐*α* in culture and measured mesenchymal transition by microarray and quantitative reverse transcription polymerase chain reaction (qRT‐PCR) analysis.^[^ ^35^ ^]^ In vitro suspension, adherent, or coated GSC cultures are often accompanied by genotypic and phenotypic changes including loss of ability to form tumors in mice.^[^ ^135^ ^]^ To mimic the in vivo environment, more studies are now focused on using 3D cultures, reflecting glioma physiology including cellular plasticity, invasion, migration, and MES transition.^[^ ^136^, ^137^, ^138^ ^]^ Since the brain TME plays an important role in GBM plasticity, patient‐derived xenograft (PDX) mouse models and patients samples organoids are more appropriate models. In an attempt to identify phenotypic plasticity in GBM, Wang et al. performed longitudinal transcriptomic profile of matched samples from primary and recurrent tumors and revealed that MES transformation occurred during recurrence.^[^ ^139^ ^]^ Neftel and colleagues used PDX models and reported that cellular states transition are common in tumors originating from a single cell clone.^[^ ^26^ ^]^

**Figure 4:**
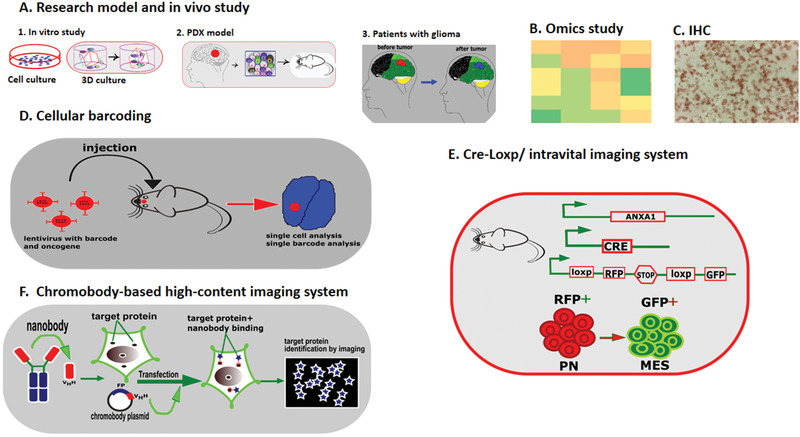
Summary of available tools/strategies to study MES signature/transition in GBM. A) Mesenchymal signature are typically evaluated by in vitro (2D and 3D culture) and in vivo (PDX and patient tissues) models; B,C) Omics (transcriptomics and proteomics) and immunohistochemistry (IHC) are gold standard tests to study cellular heterogeneity in GBM. Different assays to assess GBM MES signature: D) cellular barcoding where each transformed cell harbor a unique and heritable genetic tag is analyzed by single‐cell analysis; E) Cre‐Loxp system where Cre is cloned under a MES‐specific promoter and upon MES transition and Cre expression, cells switch from red to green; F) chromobody‐based imaging where a MES‐specific nanobody is fused to a reporter gene (chromobody) and is expressed in the cells upon MES transition.

Transcriptomic analysis on cultured cells from patient‐derived tissues and PDX is by far the most widely used model to identify cellular heterogeneity and plasticity in GBM.^[^ ^12^, ^26^, ^31^, ^139^ ^]^ Immunohistochemistry could be used to identify mesenchymal transition associated with immune infiltration,^[^ ^94^, ^95^ ^]^ while cellular barcoding combined with single cell RNAseq to monitor PMT.^[^ ^26^ ^]^ In vivo tracking of mesenchymal transition can be achieved using the Cre‐Lox and chromobody‐based imaging system.^[^ ^140^, ^141^ ^]^ Here, a Cre suitable fluorescent protein under the control of mesenchymal‐specific marker is developed in mice where a fluorescent shift is imaged (e.g., using intravital two photon microscopy) in response to mesenchymal transition.^[^ ^140^ ^]^ Chromobodies are cellular biosensors developed by fusing small‐domain antibody fragment (known as nanobodies) with a fluorescent protein, which can be detected by conventional imaging platform (e.g., immunofluorescence) and allow tracking of endogenous proteins in their native surroundings in real time.^[^ ^141^ ^]^ By identifying a specific chromobody for mesenchymal transition, one can track this process in vivo. **Figure** **4** summarizes the most exciting available tools and strategies to monitor mesenchymal transformation in GBM.

## 6 Conclusion and Perspective

Although our knowledge in GBM pathogenesis and treatment has improved drastically over the last two decades, this malignant tumor remains to be incurable. The cause behind this treatment failure is multidimensional, one of which is related to tumor heterogeneity along with phenotypic plasticity. Integration of in‐depth genomic analysis with improved preclinical models has identified morphologically distinct GBM subtypes characterized by unique genetic alterations. MES subtype among those is considered the most lethal as both preclinical and clinical studies have shown its relationship with poor prognosis and aggressiveness. In addition, subtype shifting over disease progression is a common phenomenon in GBM, and mesenchymal transformation is associated with treatment resistance. In this review, we have discussed recent advancement in mesenchymal transformation in GBM and their clinical relevance; however, before this cellular plasticity can be established as a key target for GBM adjuvant therapy, additional studies are required to clear several controversies over MES transition as reported by several investigations. For instance, some studies have suggested that mesenchymal transition is actually a two‐way phenomenon where MES GBMs can shift in the opposite direction to the PN subtype over disease progression.^[^ ^14^, ^31^ ^]^ Recent study demonstrated that each GBM subtype can robustly generate tumors in mice, while multiple subtypes can develop from a single tumor state.^[^ ^26^ ^]^ These studies the idea that mesenchymal transformation may serve as a sole target for GBM treatment. Moreover, data related to the role of TME in MES transformation, especially the immune microenvironment and response to immunotherapy, remains to be understudied and need to be further established. Finally, developing therapeutics that targets MES transformation and that can cross the blood–brain barrier and more importantly, the blood–tumor barrier, are key to prove the potential bench‐to‐bedside translation for efficient adjuvant GBM therapy.

## Conflict of Interest

The authors declare no conflict of interest.
